# Metastatic Vascular Pleomorphic Leiomyosarcoma in a Previously Treated Cervical Carcinoma Patient: A Diagnostic Dilemma

**DOI:** 10.7759/cureus.16182

**Published:** 2021-07-04

**Authors:** Umar Hakimin M Ghani, Juzaily F Leong, Mohamed H Sani, Nurwahyuna Rosli, Nor Hazla Mohd-Haflah

**Affiliations:** 1 Orthopaedics and Traumatology, Faculty of Medicine, Universiti Kebangsaan Malaysia, Kuala Lumpur, MYS; 2 Pathology, Faculty of Medicine, Universiti Kebangsaan Malaysia, Kuala Lumpur, MYS

**Keywords:** vascular leiomyosarcoma, smooth muscle connective tissue, malignant tumour, skeletal reconstruction, radiotherapy (rt)

## Abstract

Vascular leiomyosarcoma is a rare malignant tumour of the smooth muscle connective tissue. Patients are usually asymptomatic in the early stages and only present when the lesion causes compressive or obstructive effects or has metastasized. We report a case of vascular pleomorphic leiomyosarcoma in a 70-year-old lady with a background history of squamous cell carcinoma of the cervix. She presented with a three-month history of low back pain, which radiated to the anterior bilateral thigh. Initial radiological findings revealed metastatic lesions involving the spine and lungs. Two spinal biopsies done were inconclusive. Increasing severity of pain over the right thigh prompted further imaging, which revealed bilateral femoral lesions. The patient underwent surgery which involved excision of the tumour in the right proximal femur with skeletal reconstruction using megaprosthesis. Proximal femoral nail was performed for the left femur. Intra-operatively, tumour was noted at the anteromedial aspect of the proximal right thigh surrounding the superficial femoral vein. Histopathological report of the right thigh mass finally confirmed a diagnosis of vascular pleomorphic leiomyosarcoma. The patient presented four months later with bilateral pulmonary embolism with deep vein thrombosis in addition to progression of the disease.

## Introduction

Vascular leiomyosarcoma is a rare malignant tumour of the smooth muscle connective tissue. It is usually aggressive and has a high metastasis rate [1-6]. Venous leiomyosarcomas account for less than 2% of all leiomyosarcoma and commonly arise from the inferior vena cava, pulmonary vein, femoral vein, great saphenous vein, and jugular vein [1]. Delay in diagnosis is common, further attributing to its poor prognosis [2]. Chemotherapy and/or radiation therapy can improve the prognosis, but their efficacy remains debatable. Complete resection of the tumor is necessary for long-term survival [1]. In this report, we present a case of metastatic vascular pleomorphic leiomyosarcoma in a patient who was previously diagnosed with cervical carcinoma. This patient presented as a diagnostic challenge due to the late presentation of the primary lesion and unsatisfactory multiple biopsy samples. 

## Case presentation

A 70-year-old lady presented with a three-month history of low back pain radiating to the bilateral anterior thigh. Progressive worsening of her pain caused difficulty in standing and ambulation. However, there was no associated numbness in the lower limbs, neither were there any disturbances to her bowel and bladder function to indicate cauda equina syndrome. There was a history of unspecified amount of weight loss over the last six months. Further history revealed the patient had suffered squamous cell carcinoma of the cervix 20 years ago and hysterectomy was performed. Currently, she did not report any postmenopausal vaginal bleeding, pelvic or abdominal pain or anything else to suggest recurrence of the disease. On physical examination, there was tenderness over the thoracolumbar junction vertebrae region. There was good muscle power in all four limbs with preservation of sensation and tendon jerk. Gynecological examination and systemic examination were otherwise unremarkable.

Routine blood investigations and tumor markers were within normal range. MRI of the spine showed a multilevel lytic lesion with compression fracture of the T12 vertebrae. CT scan showed metastatic lungs nodules, but no evidence of pelvic mass to suggest local recurrence of cervical tumor. The provisional diagnosis at this point was metastasis of the spine and lungs with an unknown primary.

**Figure 1:**
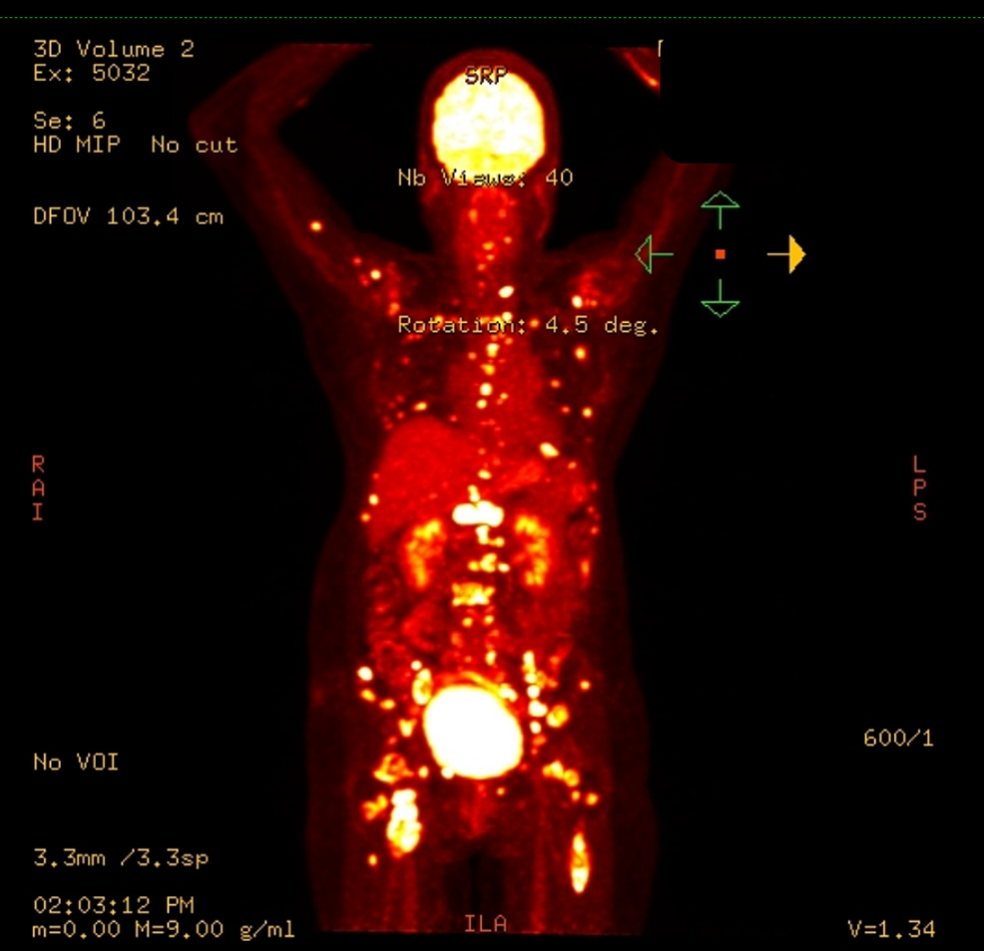
Positron emission tomography (PET) scan showed numerous hypermetabolic foci involving the entire skeleton and hypermetabolic right thigh mass.

CT-guided needle biopsies performed at the lytic lesions of the vertebrae at the level of T12 and L3 demonstrated spindle-shaped neoplastic cells of soft tissue origin but lack any malignant feature to suggest a sarcoma. A repeat biopsy was performed at a different level which was again inconclusive. PAP smear did not report recurrence of cervical malignancy. Due to difficulty in obtaining a diagnosis, positron emission tomography (PET) scan was performed to aid us in finding a primary source (Figure 1). The scan revealed numerous hypermetabolic foci involving the entire skeleton as well as a few enlarged hypermetabolic right femoral nodes.

**Figure 2:**
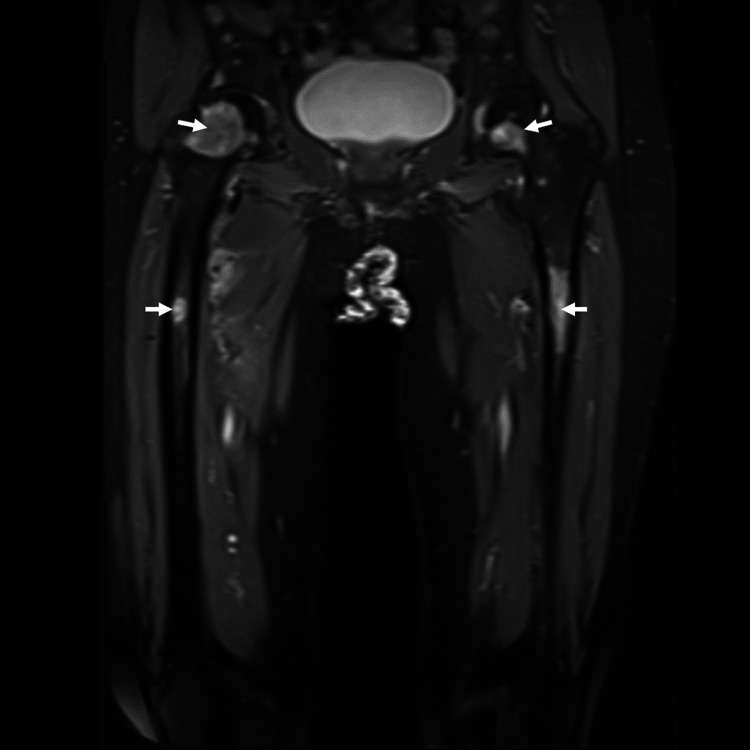
White arrows indicate extensive metastasis foci over bilateral femur.

**Figure 3:**
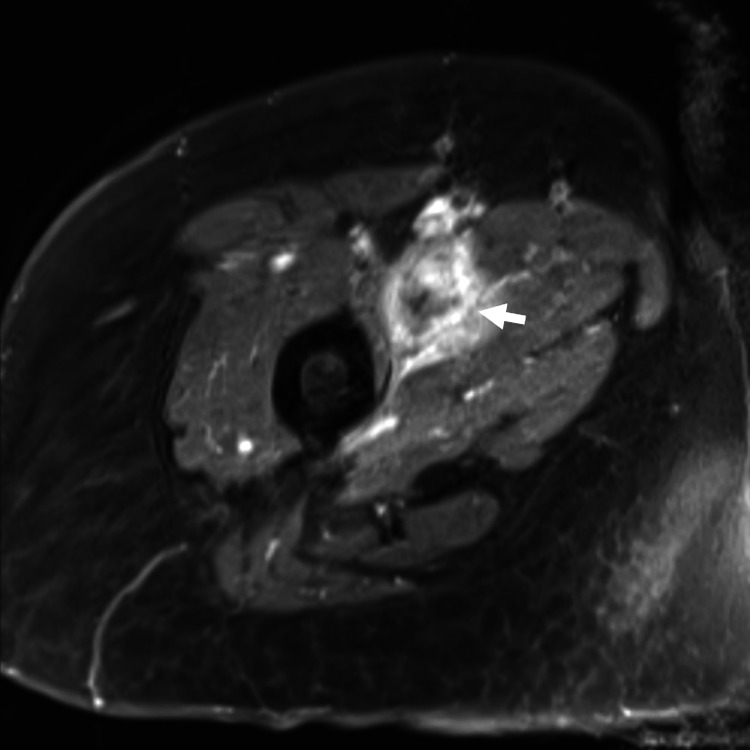
MRI of right thigh showed heterogeneously enhancing mass at the anteromedial aspect of the right thigh as shown on the white arrow.

The patient was complaining of worsening bilateral thigh pain. Right thigh examination revealed a soft tissue swelling measuring 8x6 cm at the anteromedial aspect of the thigh. MRI of the bilateral femur showed multiple lesions of both femurs (Figure 2) and a heterogeneously enhancing mass at the anteromedial aspect of the thigh encasing the right superficial femoral vein (Figure 3). Our initial opinion was that the soft tissue mass was an extension of femoral metastatic disease though we could not exclude the possibility of a soft tissue sarcoma.

**Figure 4:**
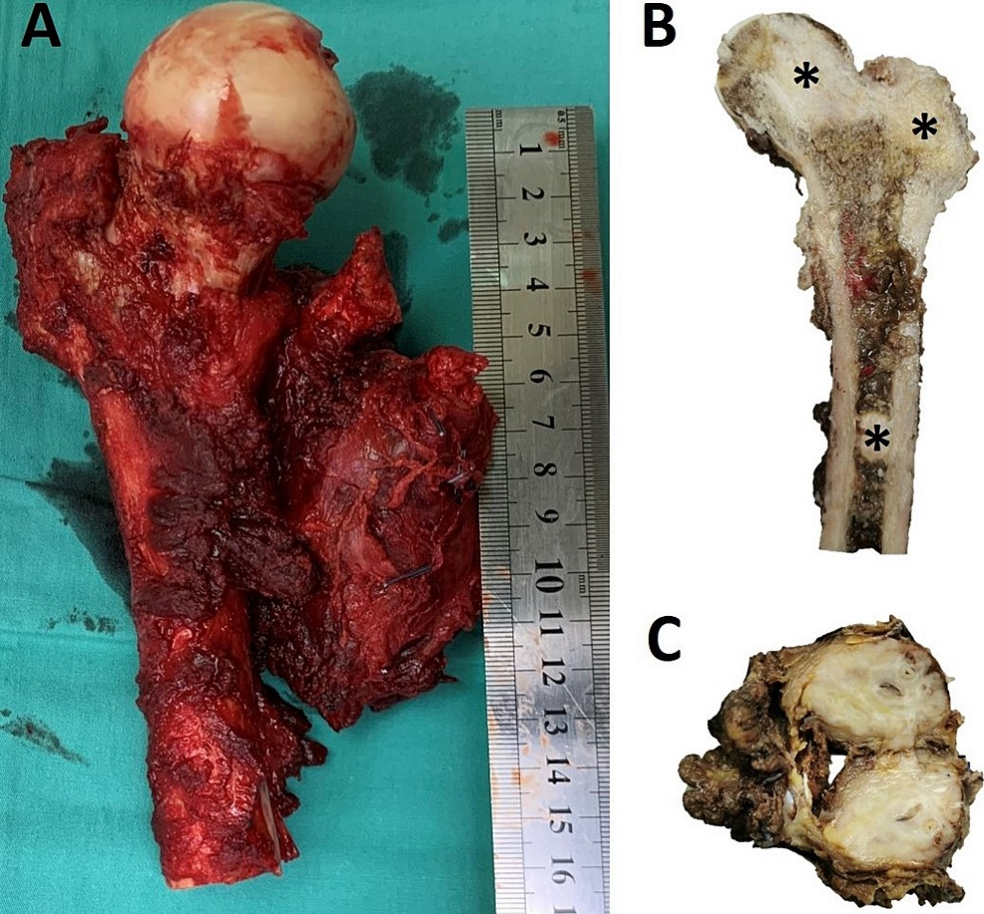
A. Excised right thigh mass and proximal right femur, B. Cut section shows irregular whitish masses (black asterisks) located at the head of femur, greater trochanter, and shaft of femur, C. Cut section of the tumour shows a well circumscribed tan whitish solid mass.

**Figure 5:**
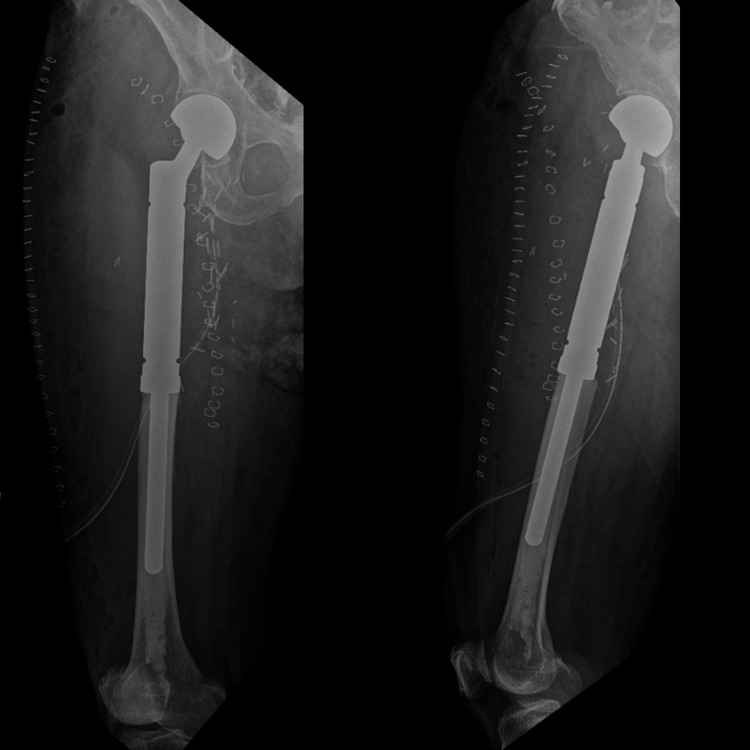
Post-operative plain radiography showing right proximal femur mega prosthesis

**Figure 6:**
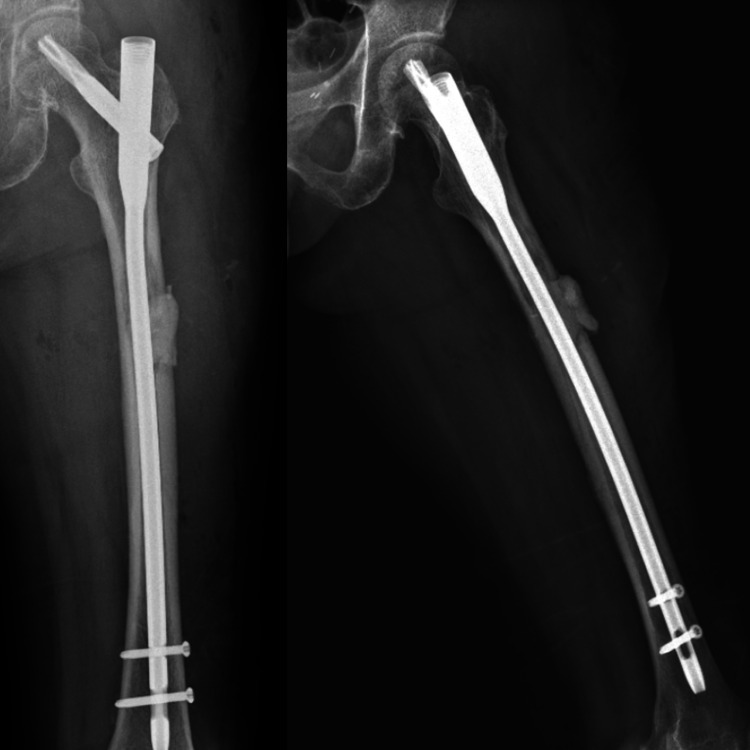
Post-operative plain radiography showing left proximal femur nail implant

At this point, despite the failure to obtain tissue diagnosis, a decision was made to perform surgery for pain relief. No tissue biopsy was performed at the thigh prior to surgery as it was apparent that whatever the diagnosis, the treatment would be palliative. Surgery would also allow us to obtain more samples for histopathological examination to aid our oncologist in determining adjuvant therapy. Due to extent of the disease in the right proximal femur, excision of the right thigh tumour (Figure 4) and skeletal reconstruction with proximal femoral megaprosthesis were performed (Figure 5). Metastatic lesion of the left femur was curetted and osteosynthesis of the proximal femoral nail with cement to cover the lesion (Figure 6). Intra-operatively, tumour was noted at the anteromedial aspect of the proximal right thigh surrounding the superficial femoral vein.

**Figure 7:**
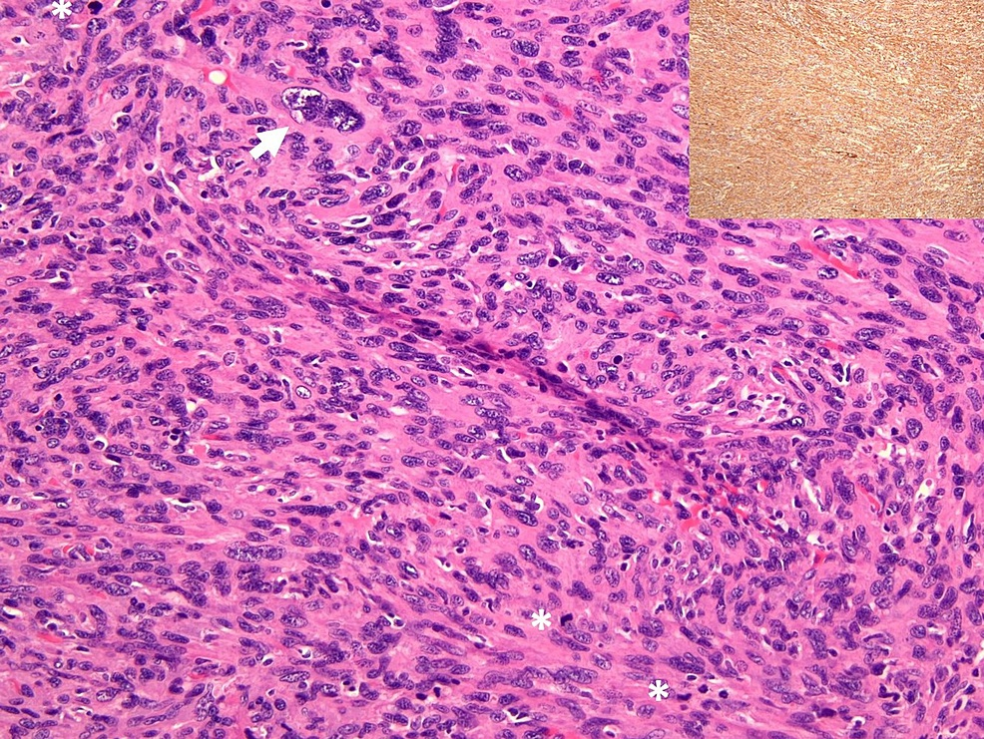
Microscopic examination shows spindle neoplastic cells exhibiting moderate to marked pleomorphism, round to plump, blunt-ended nuclei, vesicular to hyperchromatic nuclei, some with prominent nucleoli and abundant eosinophilic cytoplasm. Bizarre cells with multinucleation (white arrow) and many mitoses (white asterisks) seen (H&E, 100x). The neoplastic cells are positive for SMA (inset).

Microscopic examination revealed hypercellular tumour composed of spindle-shaped neoplastic cells arranged in intersecting fascicles (Figure 7). The spindled neoplastic cells exhibit moderate to marked pleomorphism, round to plump, blunt-ended, vesicular to hyperchromatic nuclei, some with prominent nucleoli and abundant eosinophilic cytoplasm. Occasional bizarre cells with enlarged multinucleation were also noted. Mitotic figures and tumor necrosis were present. Immunohistochemically, the neoplastic cells were positive for SMA and focally for desmin. The neoplastic cells infiltrated the surrounding skeletal muscles and adipose tissue. The final diagnosis based on histopathological examination of the right thigh mass was pleomorphic leiomyosarcoma, likely of vascular origin with distant metastasis.

The spinal metastasis was treated conservatively with a brace. Subsequently, the patient underwent postoperative adjuvant radiotherapy over the right femur and spine. Four months post-operatively, she developed bilateral pulmonary embolism with right lower limb deep vein thrombosis. CT pulmonary angiography also noted worsening lung and bone metastasis with a new right hilar lymph node, in keeping with disease progression. She is currently wheelchair-bound and receiving palliative medical treatment.

## Discussion

Vascular leiomyosarcomas (vLMS) are rare. In an early study by Italiano et al., leiomyosarcoma accounted for 13% of all soft tissue sarcoma in their centre. From this only 14% was vLMS, whereby, the majority was of venous origin [1]. Vascular leiomyosarcomas are usually high grade with poorer metastatic free survival and overall survival rate in comparison to leiomyosarcomas of other origin [1,2]. The most common location of vLMS is in the large vessels such as the inferior vena cava [1,3]. In their series, Roland et al. found that the incidence of vLMS in lower limb was only 14% of all vLMS [3]. However, Abed et al. reported the incidence of vLMS was 5.8% from all soft tissue LMS and all vLMS were located in the lower limbs [2]. In our case, intra-operatively the tumour was noted to arise from a tributary of the femoral vein. The affected vein was thrombosed with tumour tissues. Immunohistochemistry study for smooth muscle actin (SMA) and muscle-specific actin (MSA) markers are positive in the majority of histopathological samples [4].

Vascular leiomyosarcoma may present late due to their deep and intravascular location resulting in delay of diagnosis [2,5]. The development of vLMS can be divided into non-occlusive, occlusive and terminal stage. In the early stages, patients may not display any symptoms especially if there are sufficient collaterals. Tumours located in large vessels such as the inferior vena cave may grow to large size before causing occlusive or compressive effects in comparison to those arising from smaller vessels such as those in the extremities [5]. Similarly, our case lacked any clues to indicate the diagnosis of a soft tissue sarcoma in the lower limb. The presence of multiple spinal and lung metastasis with a background of cervical carcinoma, albeit 20 years ago, made primary carcinoma a more likely diagnosis. Our opinion did not differ even after obtaining MRI which demonstrated bilateral femoral involvement of the tumour. The soft tissue mass arising from the right proximal femur was assumed to be an extension of the metastatic disease. Furthermore, the size of the mass was not significant to alert us to the possibility of a soft tissue sarcoma. Abed et al. in their series of vLMS in the lower extremities observed that there was a delay in diagnosis in approximately 50% of their patients and the most common presentation in these patients was leg swelling. Routine investigations to rule out deep vein thrombosis revealed the presence of the vLMS [2]. Aside from the characteristic and location of the tumour, delay in diagnosis can also be attributed to the rare incidence of this tumour creating a lack of awareness.

The incidence of metastasis is high in vLMS, with a reported rate of up to 80%, with lung and liver being the most common site [1,4,6]. Farshid et al. stated that within a follow-up period of 36 months, 45% of patients either developed a metastatic tumour or died of the disease [6]. Abed reported that half of their patients with vLMS presented with metastasis and 75% had metastatic disease at three years of diagnosis indicating the aggressive nature of this sarcoma [2]. The angiocentricity of the tumour would be the obvious explanation of this high metastatic rate compounded by a delay in diagnosis as discussed earlier. Farshid et al. reported that the most significant risk factor for metastasis in their study was the “disruption of the tumour” which in essence indicates the margins of tumour excision. Disruption of tumour during surgery may be related to the depth of the tumour. However, they argued that other soft tissue sarcomas with similar depth have a less aggressive course. It was postulated that manipulation of the tumour during surgery could explain the high metastatic rate of vLMS [6].

For local disease, wide resection is advocated [5]. Inadvertently, this would involve resection or ligation of the affected vein. In the lower limbs, the accompanying artery can be spared or reconstructed if affected by the disease, thus circumventing the need for amputation. Reconstruction of the vein would be dictated by adequacy of collaterals [2,5]. In the lower limbs reconstruction of the femoral vein may not be required due to the presence of saphenous veins and its collaterals. The role of adjuvant chemotherapy and radiotherapy in local disease is difficult to ascertain due to the paucity of data on this disease although favourable outcomes have been reported [3]. For metastatic disease some centers have studied anthracycline-based chemotherapy regimens [1,3]. Further studies with larger sizes are required before any consensus can be made.

## Conclusions

Vascular leiomyosarcomas are aggressive malignant tumours with high recurrence rates and poor prognosis. In our case, history of previous ovarian carcinoma and inconclusive biopsy from the metastatic site resulted in delay of diagnosis, which was further compounded by the rare occurrence of this tumour. Greater awareness and knowledge about this condition can lead to prompt diagnosis and effective management of the disease.
